# Effects of mannan oligosaccharide, *Bacillus clausii*, and their synbiotic supplementation on growth performance, humoral immunity, intestinal morphology, and ghrelin gene expression in broiler chickens

**DOI:** 10.1016/j.psj.2025.105862

**Published:** 2025-09-19

**Authors:** Hanan Al-Khalaifah, Muhammad Mushtaq, Azizullah Khan, Naila Chand, Said Sajjad Ali Shah, Muqadar Shah, Asad Sultan, Rifat Ullah Khan, Shabana Naz, Ala Abudabos, Ibrahim A. Alhidary

**Affiliations:** a Environment and Life Sciences Research Center, Kuwait Institute for Scientific Research, Safat, Kuwait; b Poultry Science, Faculty of Animal Husbandry and Veterinary Sciences, The University of Agriculture Peshawar, Pakistan; c Veterinary Research Institute, Peshawar, Pakistan; d College of Veterinary Sciences, Faculty of Animal Husbandry and Veterinary Sciences, The University of Agriculture Peshawar, Pakistan; e Department of Zoology, Government College University, Faisalabad, Pakistan; f Department of Food and Animal Sciences, College of Agriculture, Tennessee State University, Nashville, TN 37209, USA; g Department of Animal Production, College of Food and Agriculture, King Saud University, Riadh, Saudi Arabia

**Keywords:** Synbiotic supplementation, Immunoglobulin response, Antibody titers, Intestinal villus architecture, Appetite-regulating hormones

## Abstract

This study evaluated the effects of a prebiotic (mannan-oligosaccharide, MOS), a probiotic (*Bacillus clausii*), and their combinations on growth performance, immune response, intestinal morphology, and ghrelin gene expression in broiler chickens. Two hundred fifty day-old Hubbard chicks were randomly allotted to five treatments (control, prebiotic, probiotic, Combo-I 50:50, Combo-II 100:100), each with five replicates of 10 birds. Combo-II supplementation improved body-weight gain and feed conversion ratio during the starter (p = 0.0256 and 0.0428), grower (p = 0.0425 and 0.0483), and finisher phases (all p < 0.05). Newcastle disease and infectious bursal disease antibody titers increased (p = 0.0445 and 0.0456) and mortality declined (p = 0.0428) in all supplemented groups, with zero mortality in the probiotic and combination treatments. Serum IgM, IgA, and IgG concentrations were elevated (p < 0.05), and duodenal villus height, villus width, crypt depth, and villus-height/crypt-depth ratio were significantly improved (all p < 0.05) in Combo-II birds. Ghrelin mRNA expression was up-regulated in pancreas and proventriculus (p < 0.05), peaking in Combo-II. These results demonstrate that full-dose prebiotic + probiotic supplementation optimizes broiler growth, immunity, gut morphology, and metabolic gene expression.

## Introduction

The global poultry industry is a key contributor to food security, providing affordable, high-quality animal protein. Despite genetic, nutritional, and management advances that have improved broiler productivity (Basit et al., 2023; Bülbül et al., 2023; Javed et al., 2023; Dinasarki et al., 2024), the routine use of antibiotic growth promoters (AGPs) has raised concerns about antimicrobial resistance, prompting regulatory restrictions and the search for safer alternatives (Magnoli et al., 2024).

Prebiotics, probiotics, and their combinations (synbiotics) are leading candidates to replace AGPs because they enhance gut health, immunity, and growth performance (Atalla et al., 2023; Tangthirasunun et al., 2024). Prebiotics are non-digestible oligosaccharides that selectively stimulate beneficial microbes and improve nutrient absorption (Devi and Mirnawati, 2023; Alqahtaani et al., 2024; Fathanah et al., 2024; Yu et al., 2024). Probiotics—live microorganisms that confer health benefits—can stabilize intestinal microbiota and modulate host immunity, and their combination with prebiotics provides additive effects (Coniglio et al., 2023).

A growing research focus is the interaction of these additives with gut-derived hormones such as ghrelin, a key regulator of feed intake, energy balance, and gastrointestinal motility (Honda et al., 2017; Gilani et al., 2021; Liang-liang et al., 2023). Although ghrelin generally stimulates appetite in mammals, avian studies indicate a more complex role, including possible suppression of feed intake and modulation of gut development (Poher et al., 2018; Mahdavi et al., 2025). Dietary prebiotics and probiotics can influence ghrelin expression, linking microbial modulation to metabolic outcomes (Gilani et al., 2021; Srifani et al., 2024; Mahdavi et al., 2025).

This study therefore evaluated the effects of dietary mannan oligosaccharide, *Bacillus clausii*, and their combinations on growth performance, immune responses, intestinal morphology, and ghrelin gene expression in broiler chickens.

## Materials and methods

### Broiler rearing and experimental treatments

A total of 250 day-old broiler (Hubbard) chicks were obtained from a local commercial hatchery. Chicks with uniform body weight were carefully selected and randomly allocated into five treatment groups. Each group was further divided into five replicates, comprising 10 chicks per replicate. The birds were reared in floor pens with a 2-inch layer of clean, dry litter material, and each replicate was housed separately to avoid cross-contamination. During the first week of brooding, an initial temperature of 32–34°C was maintained using infrared heat lamps. The temperature was gradually reduced by 2–3°C per week to reach approximately 24°C by the end of the third week, in accordance with standard brooding protocols. Continuous lighting (24 hours) was provided during the first 3 days to help chicks adjust to the environment, followed by 23 hours of light and 1 hour of darkness throughout the remainder of the trial. Adequate ventilation and controlled environmental conditions were ensured in an open-sided poultry house to maintain bird comfort. All birds were fed diets formulated according to NRC (1994) recommendations, with a starter diet provided during the starter phase (1–10 days), a grower diet during the grower phase (11–28 days), and a finisher diet during the finisher phase (29–42 days) as shown in Table 1. The experimental trial lasted for a total of six weeks. The experimental trial lasted for a total of six weeks. The control group (C) received a basal diet without any additives. The remaining four groups were supplemented as follows: the prebiotic group received mannan-oligosaccharide (MOS, 200 mg/kg of feed) derived from the cell wall of *Saccharomyces cerevisiae* (commercial product Bio-MOS®, Alltech Inc., Nicholasville, KY, USA; ≥ 90 % purity). According to the manufacturer’s certificate of analysis, this preparation contained approximately 70 % water-soluble MOS and 30 % insoluble fractions. The probiotic group received *Bacillus clausii* at 0.03 mL/kg of feed (2 × 10⁸ CFU/mL). The Combo-I group received a 50:50 quantitative combination of the prebiotic and probiotic, and the Combo-II group received a 100:100 quantitative combination of both supplements.

**Table 1:** Broiler diet formulations and nutrient composition (National Research Council NRC 1994).

Ingredients (%)	Starter (1–10 d)	Grower (11–28 d)	Finisher (29–42 d)
Maize	52.00	55.00	60.00
Soybean meal (44 %)	38.00	35.00	30.00
Vegetable oil	2.50	3.00	3.50
Dicalcium phosphate	1.80	1.60	1.40
Limestone	1.00	1.00	1.00
DL-Methionine	0.25	0.20	0.15
L-Lysine HCl	0.20	0.15	0.10
Salt	0.30	0.30	0.30
Vitamin-mineral premix¹	0.50	0.50	0.50
Total	100.00	100.00	100.00
Calculated chemical composition			
Nutrient	Starter (1–10 d)	Grower (11–28 d)	Finisher (29–42 d)
Metabolizable Energy (kcal/kg)	3000.00	3100.00	3200.00
Crude Protein (%)	22.00	20.00	18.00
Calcium (%)	1.00	0.90	0.85
Available Phosphorus (%)	0.45	0.40	0.35
Lysine (%)	1.20	1.05	0.90
Methionine + Cystine (%)	0.90	0.80	0.70

¹Vitamin-mineral premix provides per kg of diet: Vitamin A (12,000 IU), D3 (2,000 IU), E (10 mg), K (3 mg), B1 (2 mg), B2 (6 mg), B6 (3 mg), B12 (0.02 mg), niacin (40 mg), folic acid (1 mg), pantothenic acid (10 mg), biotin (0.1 mg), choline chloride (500 mg), iron (60 mg), zinc (60 mg), manganese (60 mg), copper (5 mg), iodine (1 mg), selenium (0.2 mg).

Feed and fresh drinking water were provided *ad libitum*, and separate feeders and drinkers were assigned to each replicate. All birds were managed under standard commercial conditions with adherence to routine biosecurity, hygiene, and vaccination protocols to ensure flock health and consistency across treatments.

### Zootechnical parameters

All performance indicators—including feed intake, water intake, body-weight gain, and feed conversion ratio (FCR)—were systematically recorded **at the end of each production phase (starter: days 1–10, grower: days 11–28, and finisher: days 29–42)**. Dressing percentage and carcass yield were calculated at the conclusion of the 42-day trial.

### Antibody titers

On day 30, 1 mL of blood was collected from 3 birds/replicate. Serum was separated and ND antibody titers were determined using hemagglutination inhibition (HI) test, while IBD titers were assessed using ELISA. Using V-bottom 96-well plates, serum samples were serially diluted in PBS, mixed with 4 HA units of antigen, and incubated with RBC suspension. Absence of haemagglutination (pellet formation) indicated presence of antibodies. HI titer was recorded as the reciprocal of the highest dilution inhibiting haemagglutination, expressed as log₂X (Khan et al., 2025).

### Immunoglobulin estimation

Fifteen mL blood from sheep was collected in EDTA tubes, washed with PBS, and SRBCs were prepared at 0.25 % in PBS. At 21 days, birds were injected with 0.1 mL SRBCs. After 14 days, blood was collected, serum isolated, and antibody titers measured. A direct hemagglutination assay was used to measure total antibodies (IgM, IgA, IgG). Serum was heat-inactivated (56°C, 30 min), serially diluted in PBS-BSA, and mixed with 1 % SRBCs. Plates were incubated (37°C, 24 hrs), and titers read at 50 % agglutination. For IgM/IgG differentiation, serum was treated with 0.2 M 2-mercaptoethanol (2-ME, 37°C, 30 min). Hemagglutination post-treatment indicated IgG; the difference from total titer represented IgM.

### Gut morphology and histological procedure

At the end of the experimental period, feed was withdrawn overnight from selected birds. The birds were weighed and **humanely euthanized by cervical dislocation in accordance with the AVMA Guidelines for the Euthanasia of Animals (2020)**. A 2-cm segment from the midpoint of the duodenum was excised, rinsed with physiological saline, and preserved in 10 % neutral buffered formalin. Tissue samples were processed and stained following the standard protocol described by Bancroft and Gamble, with modifications based on Zakariah et al. Fixed tissues were dehydrated, embedded in paraffin, sectioned at 5 μm thickness using a microtome, mounted on glass slides, and stained with hematoxylin and eosin (H&E). Villus height (VH) and crypt depth (CD) were measured using a light microscope under calibrated magnifications. A minimum of 10 well-oriented villi and crypts per sample were evaluated to obtain mean values (Mushtaq et al., 2025).

### Ghrelin gene expression analysis via RT-PCR

Approximately 100 mg of broiler proventriculus and pancreas tissues were collected and homogenized in TRIzol reagent for total RNA extraction, following the manufacturer’s instructions. RNA purity and concentration were measured using a NanoDrop spectrophotometer (A260/A280 ratio), and integrity was confirmed on 1 % agarose gels. To eliminate potential genomic DNA contamination, RNA was treated with DNase I (Ambion DNA-free kit). Samples were stored at −80°C until further analysis.

We validated three candidate mRNA reference genes—GAPDH, ACTB (β-actin), and RPL4—together with 18S rRNA. Expression stability across tissues and treatments was evaluated using geNorm and NormFinder, and the geometric mean of the two most stable genes (GAPDH and RPL4) was used for normalization.

Reverse transcription was carried out using the RevertAid First Strand cDNA Synthesis Kit (**Thermo Fisher Scientific**) in a final volume of 20 µL containing **0.5–1** **µg of total RNA**, 2 µL gene-specific primers (20 pmol), and DEPC-treated water to 12 µL. The mixture was incubated at 65°C for 5 min, followed by addition of the RT components and incubation at 42°C for 60 min, with a final step at 70°C for 5 min to terminate the reaction.

Quantitative PCR was performed on **Applied Biosystems StepOnePlus** using SYBR Green chemistry. Cycling conditions were: **initial denaturation at 95**°**C for 10** **min; 40 cycles of 95**°**C for 15** **s and 61**°**C for 1** **min.** Amplification efficiencies were determined from five-point standard curves and ranged from **90 % to 105 %. Ghrelin gene expression was normalized to the geometric mean of GAPDH and RPL4 and calculated using the 2^–ΔΔCt method.** No-template and no-RT controls were included to confirm absence of contamination, and PCR products were visualized on 1 % agarose gels and verified by Sanger sequencing.

### Statistical analysis

All data were analyzed using a **completely randomized design (CRD)**. Growth performance, immune parameters, intestinal morphology, and gene-expression data were first **tested for normality** using the **Shapiro–Wilk test** and for **homogeneity of variances** using **Levene’s test**. Only datasets that met these assumptions of normal distribution and equal variance were subjected to **one-way analysis of variance (ANOVA)** to evaluate treatment effects. When significant differences were detected (*p* < 0.05), means were separated using **Tukey’s Honest Significant Difference (HSD) test for** pairwise multiple comparisons. Results are presented as **mean ± standard error of the mean (SEM)**. For ghrelin gene expression, relative mRNA levels were calculated using the **2^–ΔΔCt method** after confirming amplification efficiency. These fold-change data were analyzed with the same ANOVA/Tukey procedure. All statistical analyses were performed in **IBM SPSS Statistics, version 21** (IBM Corp., Armonk, NY, USA), and graphical outputs were created in **GraphPad Prism, version 9.0** (GraphPad Software, San Diego, CA, USA).

## Results

### Growth performance

Dietary supplementation with prebiotic, probiotic, or their combinations significantly influenced broiler growth performance (Table 2). During the starter phase (1–10 days), body-weight gain (*p* = 0.0256) and feed conversion ratio (FCR; *p* = 0.0428) improved in the Combo-II group compared with the control, although feed intake was unaffected (*p* > 0.05). In the grower phase (11–28 days), feed intake (*p* = 0.0352), body-weight gain (*p* = 0.0425), and FCR (*p* = 0.0483) were all significantly better in supplemented birds, again with the greatest effect in Combo-II. During the finisher phase (29–42 days), significant differences were observed in feed intake, body-weight gain, and FCR (all *p* < 0.05), with Combo-II showing the most favorable values.

**Table 2:** Effect of prebiotic, probiotic and their combination on production performance in broiler chicks.

Period	Treatments	Feed Intake (g/bird)	Body weight gain (g/bird)	Feed Conversion Ratio (FCR
Starter period (g/week)1-10 days	Control (Basal Diet)	230.56	160.58^d^	1.32^a^
	Prebiotic (200mg/kg basal diet)	214.85	174.54^c^	1.23^b^
	Probiotic (0.03ml/Kg basal diet)	219.48	181.85^b^	1.20^bc^
	Combo-I (50:50)	234.84	195.71^ab^	1.19^c^
	Combo-II (100:100)	238.45	200.54^a^	1.18^c^
	P-Value	0.0645	0.0256	0.0428
	SEM	2.450	3.841	0.0303
Grower period (g/week)11-28 days	Control (Basal Diet)	1250.47^a^	971.72^c^	1.28^a^
	Prebiotic (200mg/kg basal diet)	1230.48^bc^	986.60^bc^	1.24^b^
	Probiotic (0.03ml/Kg basal diet)	1240.47^b^	998.68^b^	1.24^b^
	Combo-I (50:50)	1231.58^bc^	1000.54^ab^	1.23^bc^
	Combo-II (100:100)	1225.54^c^	1010.52^a^	1.21^c^
	P-Value	0.0352	0.0425	0.0483
	SEM	6.542	10.52	0.241
Finisher period (g/week) 29-42 days	Control (Basal Diet)	2451.51^a^	1052.47^d^	2.32^a^
	Prebiotic (200mg/kg basal diet)	2430.22^b^	1064.54^cd^	2.28^ab^
	Probiotic (0.03ml/Kg basal diet)	2435.84^b^	1078.41^c^	2.25^b^
	Combo-I (50:50)	2421.51^c^	1092.34^b^	2.21^bc^
	Combo-II (100:100)	2405.62^d^	1114.25^a^	2.15^c^
	P-Value	0.0358	0.0369	0.0253
	SEM	14.25	19.54	0.217

The means within the same column with at least one common letter, do not have significant difference (*P* > 0.05).

SEM: standard error of the means; Values are means ± SEM of **five replicates of 10 birds each (*n* = 50 birds per treatment group).**

### Humoral immune response

Humoral immunity and mortality (Table 3) were also enhanced by supplementation. Newcastle disease (ND) and infectious bursal disease (IBD) antibody titers increased across all supplemented groups (*p* = 0.0445 and *p* = 0.0456, respectively), while mortality decreased markedly (*p* = 0.0428), with the probiotic and both combination treatments preventing any deaths.

**Table 3:** Effect of prebiotic, probiotic and their combination on antibody titer and mortality percent in broiler chicks.

Treatments	ND	IBD	Mortality Percent
Control (Basal Diet)	4.5^c^	2268.24^c^	3.33^a^
Prebiotic (200mg/kg basal diet)	5.1^bc^	2592.47^bc^	3.33^a^
Probiotic (0.03ml/Kg basal diet)	5.4^b^	29.51.61^b^	0.00^b^
Combo-I (50:50)	6.1^ba^	3241.53^ab^	0.00^b^
Combo-II (100:100)	6.8^a^	3692.25^a^	0.00^b^
P-Value	0.0445	0.0456	0.0428
SEM	1.241	5.745	0.0113

The means within the same column with at least one common letter, do not have significant difference (*P* > 0.05).

SEM: standard error of the means.

values are means ± SEM of **10 birds per treatment (2 birds sampled per replicate pen).**

Immunoglobulin concentrations against sheep red blood cells (Table 4) rose significantly for IgM, IgA, and IgG in all supplemented treatments (*p* < 0.05), with the highest responses observed in the full-dose combination.

**Table 4:** Effect of prebiotic, probiotic and their combination on immunoglobulin titer against the sheep RBCs in broiler chicks.

Treatments	IgM	IgA	IgG
Control (Basal Diet)	1.78^c^	0.05^c^	0.04^c^
Prebiotic (200mg/kg basal diet)	1.82^bc^	0.06^c^	0.05^b^
Probiotic (0.03ml/Kg basal diet)	1.87^b^	0.08^b^	0.05^b^
Combo-I (50:50)	1.95^ab^	0.08^b^	0.06^b^
Combo-II (100:100)	2.04^a^	0.15^a^	0.08^a^
P-Value	0.0355	0.0421	0.0417
SEM	0.045	0.084	0.010

The means within the same column with at least one common letter, do not have significant difference (*P* > 0.05).

SEM: standard error of the means.

values are means ± SEM of **10 birds per treatment (2 birds sampled per replicate pen).**

### Gut histology

**Fig. 1:**
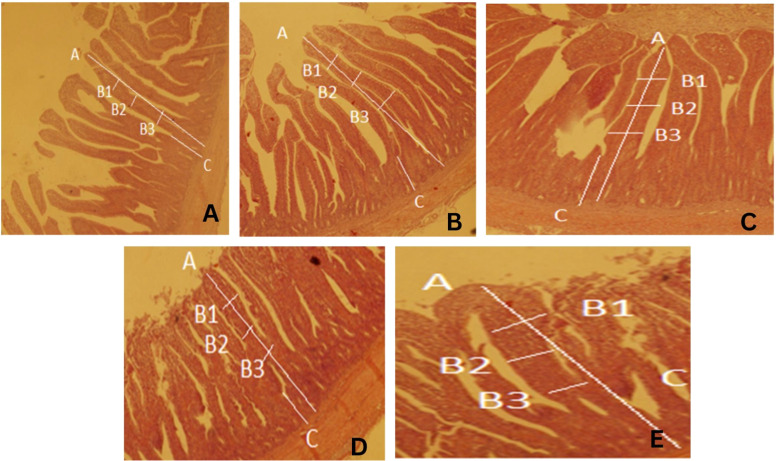
Duodenum villus morphology in Control Group (A) showing villus height (A), width (B1–B3), and crypt depth (C) at 40X, Prebiotic-fed Group (B), Probiotic-fed Group (C) Combo-I Group (D) and Combo-II Group (E).

Intestinal histomorphology (Table 5; Fig. 1) showed significant improvements in villus height, villus width, crypt depth, and villus-height-to-crypt-depth ratio (all *p* < 0.05) for the combination treatments compared with the control.

**Table 5:** Effect of prebiotic, probiotic and their combination on histomorphology of duodenum in broiler chicks.

Treatments	Villus Height (µm)	Villus width (µm)	Crypt depth (µm)	VH:CD
Control (Basal Diet)	540.12^d^	42.51^c^	102.54^a^	5.26^d^
Prebiotic (200mg/kg basal diet)	650.45^c^	69.54^bc^	96.85^ab^	6.71^c^
Probiotic (0.03ml/Kg basal diet)	690.88^bc^	80.28^b^	94.21^b^	7.33^bc^
Combo-I (50:50)	701.95^b^	84.74^b^	82.59^c^	8.49^b^
Combo-II (100:100)	759.47^a^	102.51^a^	72.51^d^	10.47^a^
P-Value	0.0254	0.0284	0.0352	0.0425
SEM	30.45	7.54	6.58	0.536

The means within the same column with at least one common letter, do not have significant difference (*P* > 0.05).

values are means ± SEM of **10 birds per treatment (2 birds sampled per replicate pen).**

### Ghrelin gene expression

**Fig. 2:**
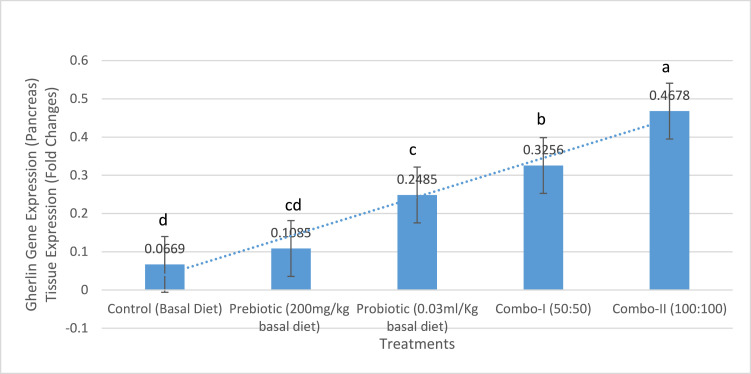
Tissue expression (fold change) of ghrelin in the pancreas of chicken broilers treated with different growth promoters and their combinations. a,b,c Within individual charts, bars representing mean gene expression with no common superscripts differ significantly (*P* < 0.05). values are means ± SEM of **10 birds per treatment (2 birds sampled per replicate pen)**.

**Fig. 3:**
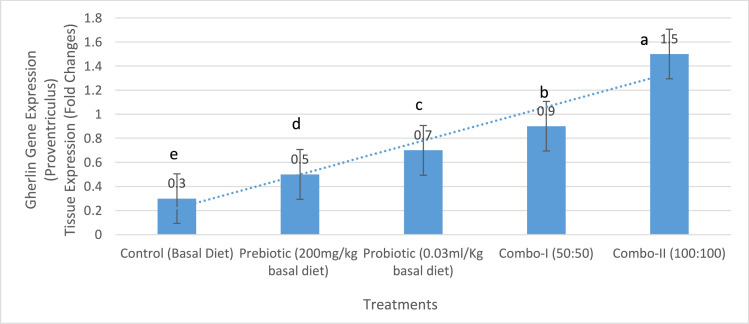
Tissue expression (fold change) of ghrelin in the proventriculus of chicken broilers treated with different growth promoters and their combinations. a,b,c Within individual charts, bars representing mean gene expression with no common superscripts differ significantly (*P* < 0.05). values are means ± SEM of **10 birds per treatment (2 birds sampled per replicate pen)**.

Ghrelin gene expression increased in a dose-dependent manner. In the pancreas (Fig. 2), expression differed significantly among treatments (*p* < 0.05), with the combination groups showing the greatest upregulation. A similar pattern occurred in the proventriculus (Fig. 3), where fold change rose progressively with increasing supplementation level (*p* < 0.05).

## Discussion

The present study demonstrated that dietary supplementation with a combination of prebiotics and probiotics (Combo-II) consistently improved growth performance parameters in broiler chicks across all growth phases. These improvements were particularly evident in body weight gain (BWG) and feed conversion ratio (FCR), while feed intake was either unaffected or slightly reduced. Such outcomes suggest that the enhanced performance was not a result of increased feed consumption, but rather **reflects improved nutrient absorption and energy partitioning toward muscle accretion** (Khan and Naz, 2013). **This indicates that the birds utilized dietary nutrients more efficiently, which is a key economic advantage in commercial broiler production (**Dalbool et al., 2024**;** Habib et al., 2024**).** These effects are likely mediated through multiple physiological and microbial mechanisms triggered by the synbiotic action of prebiotics and probiotics (Ciptaan et al., 2024; Yu et al., 2024).

Prebiotics, such as mannan-oligosaccharides, and probiotics, particularly *Lactobacillus* and *Bifidobacterium* species, play complementary roles in gut health (Obayomi et al., 2024). Prebiotics serve as selective substrates for beneficial microbes, while probiotics provide live beneficial strains that colonize the gut and suppress pathogenic bacteria (Khan et al., 2020). Their combined supplementation (synbiotics) leads to improved microbial balance in the gastrointestinal tract, creating a more favorable environment for nutrient digestion and absorption (Gulzar et al., 2019; Obayomi et al., 2024). **The present findings of increased villus height and villus-height–to–crypt-depth ratio strongly support this mechanism, demonstrating direct structural evidence of enhanced absorptive capacity.** The enhanced microbial activity promotes the production of short-chain fatty acids (SCFAs), such as butyrate and propionate, which are known to strengthen the intestinal epithelium, reduce inflammation, and stimulate enterocyte proliferation (Khan and Naz, 2013). **Consequently, a healthier gut not only facilitates better nutrient uptake but also limits the energy cost of immune activation by reducing pathogen load.**

In addition, synbiotics are known to upregulate the activity of digestive enzymes and nutrient transporter proteins, further enhancing the efficiency of nutrient assimilation (Gulzar et al., 2019). The reduced FCR observed across all phases in Combo-II birds indicates more efficient conversion of feed into body mass, likely a result of these gut-level improvements. Moreover, **this improved efficiency can reduce feed costs and lower nitrogen excretion, which is environmentally beneficial in intensive poultry systems.** Modulation of gut-associated lymphoid tissue by synbiotics may enhance mucosal immunity and overall health, reducing energy expenditure on immune responses and redirecting nutrients toward growth (Methiwala et al., 2021).

A possible contributing factor to the improved feed efficiency is the observed upregulation of ghrelin gene expression in the proventriculus of Combo-II birds (Fig. 3). Ghrelin, a gut-derived peptide hormone, plays a key role in regulating appetite, energy balance, and growth hormone release (Mahdavi et al., 2025). **Although feed intake did not increase, elevated ghrelin expression may stimulate growth hormone secretion and anabolic pathways, helping explain the superior weight gain despite stable intake.** Its increased expression may have supported better nutrient partitioning and metabolic efficiency, thereby contributing to higher BWG even when feed intake was slightly lower (Gilani et al., 2021). This suggests a gut–brain axis interaction in which improved microbial composition indirectly influences endocrine control of growth. Overall, the continuous and synergistic interaction between gut microbiota modulation, improved intestinal structure, enhanced digestive function, and endocrine regulation explains the superior growth performance observed in birds fed the Combo-II diet. These findings support the inclusion of synbiotics as an effective nutritional strategy to optimize broiler growth and feed efficiency without increasing feed consumption.

The supplementation of prebiotics, probiotics, and their combinations plays a pivotal role in enhancing the immune function of broiler chickens through multiple, well-established mechanisms (Adhikari and Kim, 2017; Akhter et al., 2023; Rashid et al., 2023b, Rashid et al., 2023a). The immunomodulatory effects of these additives stem largely from their influence on gut microbial composition, intestinal integrity, and systemic immune signaling pathways, which together contribute to a more robust humoral immune response and reduced mortality in poultry (Khan and Naz, 2013; Younas et al., 2025). Probiotics, especially lactic acid bacteria such as Lactobacillus spp., have been shown to stimulate immune cells by interacting with pattern recognition receptors (PRRs) like Toll-like receptors (TLRs) on the gut-associated lymphoid tissue (Khan et al., 2020; Nguyen et al., 2024). This interaction enhances the maturation and function of B cells, leading to increased production of immunoglobulins including IgM, IgA, and IgG. **The significantly higher antibody titers to ND and IBD observed here confirm that these pathways were activated, providing functional protection beyond vaccine responses.** Secretory IgA, in particular, is critical for mucosal defense, while IgM and IgG are involved in systemic immunity. Probiotic-driven increases in antibody titers against pathogens such as Newcastle Disease (ND) and Infectious Bursal Disease (IBD) have been reported in various studies, reflecting their capacity to enhance both mucosal and systemic humoral responses (Adhikari and Kim, 2017).

Prebiotics, such as mannan-oligosaccharides (MOS), further support immune health by selectively stimulating beneficial gut bacteria, especially *Bifidobacteria* and *Lactobacilli* (Khan et al., 2020). These beneficial microbes produce metabolites like short-chain fatty acids (SCFAs), particularly butyrate, which serve as energy sources for colonocytes and help maintain epithelial barrier integrity (Khan and Naz, 2013). A well-maintained intestinal barrier reduces pathogen translocation and endotoxin burden, allowing the immune system to function more efficiently (Younas et al., 2025). Additionally, prebiotics have been reported to enhance macrophage activity and cytokine production, thereby indirectly supporting antibody production (Adhikari and Kim, 2017). **The near-zero mortality recorded in the synbiotic groups underscores the practical relevance of these immune benefits for flock health and farm profitability.**

When administered in combination, prebiotics and probiotics (i.e., as synbiotics) exert a synergistic effect, improving colonization of beneficial microbes and amplifying immune stimulation. This synergy likely accounts for the superior immune responses observed with full-dose combinations (Adhikari and Kim, 2017). Synbiotics can modulate both innate and adaptive immunity by increasing lymphocyte proliferation and stimulating cytokines such as IL-2 and IFN-γ, which support antibody synthesis and class switching (Yadav et al., 2022). **Such coordinated innate–adaptive activation offers a non-antibiotic strategy for disease prevention, aligning with global efforts to reduce antimicrobial use in poultry production.**

Dietary supplementation with prebiotics, probiotics, and their combinations exerts significant influences on intestinal morphology, which is critical for nutrient absorption and gut health in broilers (Wang et al., 2016). Improvements in villus height, villus width, and the villus height-to-crypt depth (VH:CD) ratio, alongside reductions in crypt depth, reflect enhanced gut development and functionality. Probiotics contribute to intestinal health by modulating gut microbiota, suppressing pathogenic bacteria, and stimulating epithelial cell proliferation (Khan and Naz, 2013). These effects promote the development of longer and thicker villi, increasing the absorptive surface area of the small intestine and improving nutrient uptake (Adhikari and Kim, 2017). Additionally, probiotics produce metabolites like short-chain fatty acids and bacteriocins, which further stimulate intestinal epithelial regeneration and maintain mucosal integrity (Khan and Naz, 2013). **Our finding of a markedly higher VH:CD ratio in Combo-II birds provides histological confirmation of these processes.**

Prebiotics, such as fructo-oligosaccharides and mannan-oligosaccharides, selectively support the growth of beneficial bacteria like Lactobacillus and Bifidobacterium, enhancing microbial balance and reducing intestinal inflammation (Khan et al., 2020; Obayomi et al., 2024). By modulating local immune responses and microbial fermentation, prebiotics promote shallower crypts and taller villi, suggesting lower epithelial turnover and better nutrient utilization efficiency (Yadav and Jha, 2019). When prebiotics and probiotics are administered together as synbiotics, their synergistic effects intensify gut morphological improvements. Synbiotics enhance microbial colonization, stimulate brush-border enzyme activity, and upregulate the expression of tight junction proteins, all of which contribute to improved villus architecture and digestive function (Da Silva et al., 2021; Tong et al., 2023). **These structural enhancements explain the consistent improvements in FCR across all growth phases observed in our study.**

The significant upregulation of ghrelin gene expression in both the pancreas and proventriculus of broiler chickens following supplementation with prebiotics, probiotics, and particularly their combinations, reflects the modulatory impact of gut microbiota on neuroendocrine signaling pathways involved in energy homeostasis and appetite regulation (Gilani et al., 2021). Ghrelin, a peptide hormone primarily produced in the gastrointestinal tract, plays a key role in stimulating feed intake and promoting anabolic processes through its interaction with the growth hormone secretagogue receptor (GHS-R) in the hypothalamus (Dimaraki and, 2006). Its expression is known to be sensitive to nutritional status and microbial signals within the gut environment. The enhanced ghrelin expression observed in supplemented groups, especially with the full-dose combination (Combo-II), suggests a microbiota-mediated improvement in gut-brain axis communication (Gilani et al., 2021). Probiotics influence ghrelin expression through several mechanisms. First, by increasing populations of beneficial microbes such as Lactobacillus and Bifidobacterium, probiotics improve gut integrity and modulate enteroendocrine function, leading to increased ghrelin synthesis (Yu et al., 2024). Additionally, the metabolic byproducts of these microbes—such as short-chain fatty acids—can directly affect the transcription of ghrelin and other orexigenic peptides through epigenetic and receptor-mediated pathways (Hamamah et al., 2023). **These findings highlight a novel endocrine dimension of synbiotic action, linking gut microbiota changes to hormonal regulation of growth.**

Prebiotics, on the other hand, selectively stimulate the growth of commensal bacteria that ferment dietary fibers to produce SCFAs like butyrate and propionate (Adhikari and Kim, 2017). These SCFAs have been implicated in influencing gut hormone secretion, including ghrelin, by acting on G-protein-coupled receptors (GPCRs) such as GPR41 and GPR43 located on enteroendocrine cells (Cani et al., 2013). However, the relatively modest effect of prebiotics alone in this study suggests that microbial fermentation efficiency and subsequent hormone modulation are more potent when combined with live microbial supplementation. The synergistic effects of synbiotics (Combo-I and Combo-II) likely result from improved microbial colonization and SCFA production, leading to stronger stimulation of ghrelin-secreting cells in both the pancreas and proventriculus. This may contribute to improved appetite regulation, feed efficiency, and nutrient utilization. The enhanced ghrelin expression observed in the Combo-II group aligns with previous findings indicating that gut microbiota composition can significantly influence the expression of appetite-regulating hormones and metabolic mediators in poultry and other animals (Yu et al., 2024).

## Conclusion

Full-dose combined supplementation of **mannan-oligosaccharide (200** **mg/kg feed)** and ***Bacillus clausii* (0.03** **mL kg⁻¹ feed)** (Combo-II) produced the most pronounced benefits across all measured parameters. Birds receiving this synbiotic diet showed **greater body-weight gain and improved feed conversion ratio** throughout the starter, grower, and finisher phases (all *p* < 0.05). **Humoral immunity** was enhanced, with significantly higher Newcastle disease and infectious bursal disease antibody titers and increased serum IgM, IgA, and IgG concentrations. **Intestinal histomorphology** revealed taller villi, wider villus tips, and a higher villus-height-to-crypt-depth ratio, indicating superior nutrient absorption. Furthermore, **ghrelin gene expression in both pancreas and proventriculus was up-regulated**, pointing to improved metabolic regulation. Future studies should examine (i) the long-term effects of synbiotic use on carcass quality and meat microbiota, (ii) cost–benefit analyses under large-scale field conditions, and (iii) potential synergistic effects when combined with alternative feed additives such as phytogenic compounds or organic acids.

## Funding

Not applicable

## Ethical statement

This study was approved by the ethical committee of, the Department of Poultry Science, Faculty of Animal Husbandry and Veterinary Sciences, The University of Agriculture Peshawar, Pakistan under notification No. L- 452/AH/UAP dated 16/11/2022.

## Data availability statement

Data is available from the corresponding author upon reasonable request.

## CRediT authorship contribution statement

**Hanan Al-Khalaifah:** Funding acquisition. **Muhammad Mushtaq:** Conceptualization, Supervision. **Azizullah Khan:** Data curation, Investigation, Methodology. **Naila Chand:** Supervision. **Said Sajjad Ali Shah:** Methodology, Resources. **Muqadar Shah:** Software, Validation, Visualization. **Asad Sultan:** Resources. **Rifat Ullah Khan:** Data curation, Investigation, Methodology. **Shabana Naz:** Writing – original draft, Writing – review & editing. **Ala Abudabos:** Writing – original draft, Writing – review & editing. **Ibrahim A. Alhidary:** Writing – original draft, Writing – review & editing.

## Disclosures

Authors declare no conflict of interest
